# The Influence of Tobacco Smoking on the Onset of Periodontitis in Young Persons

**DOI:** 10.1186/1617-9625-2-6

**Published:** 2004-06-15

**Authors:** Brian H Mullally

**Affiliations:** 1 Division of Restorative Dentistry (Periodontics), School of Clinical Dentistry, Queen's University of Belfast, Northern Ireland

## Abstract

This paper reviews the evidence for cigarette smoking as a risk factor for the development of severe destructive periodontal disease in young adults. A high prevalence of cigarette smoking has been identified among young individuals with aggressive periodontitis and tobacco usage increases the risk of periodontal destruction most significantly in young populations. The effect appears to be dose related and is independent of levels of plaque accumulation. Young smokers have more alveolar bone loss and attachment loss than non smoking equivalents. Prolonged and heavy smoking can reduce gingival bleeding and therefore mask the clinical marker of bleeding on probing often used by dentists to monitor periodontal health. This has implications for potential misdiagnosis and failure to detect periodontitis at an early stage. Nicotine metabolites concentrate in the periodontal tissues and can have local effects as well as the potential to affect the systemic host response. Dentists are well placed to assess the smoking status of their young patients and have a role to play in the delivery of smoking cessation advice especially as it pertains to periodontal health. In this way the dental profession can also make a significant contribution to the general health and well being of our youth and future generations.

## Introduction

Periodontal diseases are a group of conditions affecting the supporting structures for the dentition. The periodontal tissues consist of a specialized form of oral mucosa known as gingiva, which has a keratinized epithelium and covers the alveolar bone. There is an epithelial attachment between the enamel of the tooth and the marginal gingivae which is formed from the fusion of reduced enamel epithelium and the oral epithelium and is known as junctional epithelium when tooth eruption is completed [1]. This provides the biological seal for the tooth-gum interface in the oral cavity and in health provides a barrier to potential ingress of infective organisms. A complex structure of collagen fibres attaches to the gingival tissues and provides further support for the dentition by connecting the root surface to the alveolar bone to form the periodontal ligament.

Inflammation of the marginal gingival tissues is a common condition and its extent and severity can be variable. This condition known as gingivitis can be modified by systemic and local influences and is plaque induced. Often it can be reversed if improved oral hygiene measures are introduced.

Chronic periodontitis is the result of a response of the host to bacterial aggregations on the tooth surfaces. The outcome of this is an irreversible destruction of the connective tissue attachment, which results in periodontal pocket formation and eventual loss of alveolar bone. While gingivitis is known to be a very prevalent condition among children and adolescents, periodontitis is much less common in this group. The occurrence of severe periodontitis in young adults may have a devastating effect on their dentition and in some cases treatment of these forms of periodontal disease can be unsuccessful.

Diagnosis of periodontitis and the identification of affected individuals can sometimes be difficult because there may be no self-reported symptoms. It is therefore recommended that clinicians should screen patient's susceptibility to periodontitis by evaluating their exposure to associated risk factors so that early detection and appropriate management can be achieved. Destructive periodontitis has been described as a consequence of the interaction of genetic, environmental, microbial and host factors [2].

Among those risk factors identified for periodontitis are age, gender, socioeconomic status, and genetic predisposition, bacterial colonisation, certain systemic conditions and smoking.

Tobacco smoking has been found to be a major environmental factor associated with generalized forms of severe periodontitis in several studies. As long ago as 1848, John Burdell, an American dentist, described the oral changes associated with tobacco chewing and commented on the difficulties he had experienced in providing dentures for this group. His book, *Tobacco: Its Use and Abuse*, contains a reference to gingival recession in tobacco users and the subsequent loosening of the mandibular incisor teeth [3]. Few references to the relationship between smoking and periodontal disease appear in the dental literature until almost a century later when Pindborg [4] described the association between acute ulcerative gingivitis and tobacco consumption in Danish military recruits.

Hujoel and colleagues [5] recently investigated the past and future changes in incidence of advanced periodontitis in a U.S. population aged 30–39 years. This group highlighted the deficiencies in periodontal epidemiology including the late recognition of tobacco smoking as an important risk factor for periodontitis and the inadequacy of measuring periodontal destruction in many national surveys. Hujoel suggests that a hidden periodontitis epidemic related to smoking patterns occurred during the 20th Century and that sociodemographic shifts in smoking habits will alter the periodontal needs for the future. They predict a polarization of periodontitis within populations such as lower socioeconomic groups in the U.S. and developing countries where smoking prevalence is on the increase. These investigators recommend that periodontal researchers should study never-smokers separately from those with either a current or previous smoking history.

This paper will review the evidence for cigarette smoking as a risk factor for the development of severe destructive periodontal disease in young adults.

The recent world workshop on periodontal disease classification introduced the term aggressive periodontitis [6]. Previously a variety of clinical patterns of severe periodontitis in young adults characterized by significant loss of alveolar bone and attachment have been known as early onset or juvenile periodontitis. For the purpose of this review, these terms will be considered to be interchangeable.

## Prevalence of Early onset or Aggressive Periodontitis in Young Adults

Evidence in the existing literature suggests that ethnic and genetic factors are important in the development of periodontitis in young adults. A number of studies report low prevalence rates for aggressive periodontitis in European populations. Reported prevalence rates in these groups range from 0.1–0.2% [7,8]. There is evidence that African populations are more susceptible to aggressive periodontitis. Löe and Brown [9] estimated that Black American teenagers were 15 times as likely to have early onset periodontitis, as were white Americans. Harley and Floyd, [10] reported that 0.8% of Nigerian teenagers had early onset periodontitis. Arowojolu and Nwokorie [11] found a prevalence of 1.6% in those aged 17–34 years attending a Nigerian Hospital clinic. More recently Albandar and colleagues [12] reported a high prevalence of aggressive periodontitis among 12–25 year olds attending Ugandan schools. In this study they found 6.5% of the group had either localized or generalized form of this disease and a further 22% had incidental aggressive lesions. Hodge and colleagues [13], using segregation analysis, reported on the factors associated with the occurrence of early onset periodontitis in a large European Caucasian family. In this study the investigators found that genetic factors were more significant than smoking history in the manifestation of generalized early onset periodontitis. The precise mechanism whereby cigarette smoking exerts an effect on the development of periodontal destruction is unknown.

## Smoking and Gingival Inflammation

A reduction in clinical signs of gingivitis has been reported in smokers and this effect has been shown to be independent of plaque levels [14]. There have been other reports of less bleeding in smokers with periodontitis, suggesting that nicotine could mediate its vasoactive effects on a local basis. [15,16]. It has also been demonstrated that smoking can delay the inflammatory response in reaction to experimental gingivitis [17]. Holmes and colleagues [18] demonstrated a reduction in Gingival Crevicular Fluid (GCF) flow in smokers with clinically healthy gingival tissues compared with matched non-smokers. McLaughlin and colleagues [19] reported that smoking a cigarette caused an immediate increase in GCF flow while Bergstrom and Preber [20] found that GCF flow diminished in chronic smokers. Linden and Mullally [21] found that young smokers had in fact more gingival bleeding than non-smoking regular attenders. The explanation for this finding seemed to be related to the high levels of calculus and plaque reported in this group of young adults. Other studies of older population groups have found little difference in plaque accumulation between smokers and non-smokers [17,22]. Linden and Mullally [21] reported on 82 young adults who were regular attenders at general dental practices throughout Northern Ireland. All subjects underwent a comprehensive periodontal examination and smoking history was determined by way of a questionnaire. These subjects were aged 21–33 years and 21 of the group were current smokers. There were no differences between the smokers and non-smokers with regard to plaque accumulation, although smokers had twice as many sites with calculus deposits. These young smokers had significantly more sites, which bled on probing (41%) compared with nonsmokers (29%). In a longitudinal study over a six month period, Müller and colleagues [23] observed that the potential for the transformation of non-bleeding sites to bleeding among smokers, compared with non-smoking controls in a young predominantly male military population, was increased by 86%. They also reported higher levels of supragingival plaque and calculus in smokers in their population who received no periodontal treatment during the observation period.

Clarke and colleagues [24] demonstrated an initial increase in gingival blood flow associated with nicotine consumption using a rabbit model. This was followed by a reduction to below baseline levels. Using Doppler Flowmetry, Baab and Öberg [25] observed a significant, immediate increase in gingival circulation in a group of college students during smoking. Gingival blood flow returned toward baseline within 10 minutes. His study group consisted of 12 young smokers with a daily consumption of 5–15 cigarettes who had been smokers for 2–8 years. More recently Palmer and colleagues [26] measured gingival blood flow, also using a laser Doppler technique, and their data did not support the view that smoking compromised blood flow in the periodontal tissues. The results of this study therefore cast doubt on the assumption that this was a mechanism by which individuals might be predisposed to develop periodontal disease. However as the authors concede the technique of laser Doppler flowmetry is only applicable in the measurement of acute changes in blood flow and there is still the possibility that chronic exposure to tobacco smoke might affect blood flow in the periodontal tissues. Tobacco use has also been associated with reduced permeability of peripheral blood vessels. [27]. Other studies [28] have demonstrated differences in the oxygen saturation of haemoglobin between smokers and nonsmokers, which are indicative of functional defects in the microvasculature of those who smoke. While there might be some controversy regarding the effect of tobacco consumption on the gingival vasculature, the clinical relevance is clear. Prolonged and heavy smoking can reduce gingival bleeding and therefore mask the clinical marker of bleeding on probing often used by dentists to monitor periodontal health. This has implications for potential misdiagnosis and failure to detect periodontitis at an early stage.

## Smoking and Extent of Periodontitis

Mahuca and colleagues [29] evaluated the degree of periodontal disease and its relationship to smoking habits in a population of young healthy male Spanish military recruits. They report higher plaque and bleeding indices in non-smokers although probing depths and attachment loss were greater in smokers. Young smokers diagnosed with aggressive forms of periodontitis were shown to have more affected teeth and a higher mean loss of periodontal attachment than non-smokers with these conditions [30,31].

In a clinical study of patients treated in general dental practice in Northen Ireland, Linden and Mullally [21] reported that the percentage of sites with probing depths in excess of 4 mm was more than double in young smokers (15%) compared with 6% in non-smokers. The extent of periodontitis as evaluated by the percentage of sites with attachment loss more than 2 mm was 22% for young adults who smoked compared with 9% in those who did not. The odds ratio for established periodontitis as outlined by Machtei and colleagues [32], i.e., at least 2 sites with attachment loss of 6 mm or more and one site with pocketing of 5 mm, was found to be 14.1 for cigarette smokers. Other clinical studies have confirmed that smoking is a true risk factor for periodontitis with odds ratios for periodontitis in the range of 2.0–7.0 [33].

A number of clinical investigations have reported that cigarette smokers with aggressive or early onset periodontitis have more extensive periodontal destruction in the maxillary region. [31,34]

Schenkein and colleagues [30] reported on the clinical status of subjects with varying degrees of periodontal destruction. These investigators found a higher prevalence of smoking among patients diagnosed with generalized early-onset or aggressive periodontitis and adult periodontitis than in those with localized juvenile periodontitis or with good periodontal health. For instance they reported that 20% of subjects with localised aggressive periodontitis, 43% with generalised and 16% of healthy subjects were smokers. Significant effects were also seen in relation to periodontal attachment loss in smokers with generalized early-onset periodontitis. These patients had significantly more extensive periodontitis, more teeth with affected sites, and a greater mean loss of attachment than patients who did not smoke. Thus, the risk of smoking could greatly accelerate tooth loss in this relatively young group of individuals who are already at high risk for progressive periodontal attachment loss. Smoking was found to be a major environmental factor associated with generalized forms of aggressive periodontitis in several other studies [31,35]. Several authors have reported a high prevalence of smoking among patients with aggressive periodontitis. Mullally and colleagues [31] reported that 70% of subjects with generalized and 44% of those with localized aggressive periodontitis were cigarette smokers. The smoking prevalence for individuals with generalized aggressive periodontitis was twice the population value for comparable age groups in Northern Ireland. While the effects of smoking appear to be more notable among those with generalized aggressive periodontitis, Schenkein and colleagues (1995) suggest that patients with localized juvenile periodontitis who smoke are not clinically distinguishable from non-smoking subjects with this condition. The results of other studies [31,36] would tend to support the view that cigarette smoking exerts a more significant effect on those with generalized forms of aggressive periodontitis.

Some studies have also highlighted the dose relationship between the effect of cigarette consumption and periodontal attachment loss. In a New York population of old and young subjects Zambon and colleagues [37] reported that heavy smokers were more than twice as likely to experience attachment loss and alveolar bone destruction as light smokers were. Mahuca and colleagues [29] reported more attachment loss in a young male population who were heavier smokers. These investigators also reported a relationship between the length of time that the individual had been smoking and increased clinical attachment level. Lopez [38] could find no evidence of an increase in risk of developing periodontitis in a large sample of Chilean students aged 12–21 years. However this study reported a weak dose relationship in that the highest odds for clinical attachment loss were observed in those young adults with the most smoking exposure. A later report from this research team on what appears to be the same population investigated the prevalence of Necrotizing ulcerative gingivitis and could find no relationship between this destructive periodontal condition and cigarette smoking [39]. This is in conflict with many earlier studies, in which a significant relationship was observed. In spite of their observation that cigarette smoking was habitual in 25% of this group, these researchers opined that the lack of association with disease and smoking could be accounted for by the short duration of the smoking habit (less than 3 years) and low consumption (less than 5 per day). In a study of young adults with severe periodontal destruction Mullally and colleagues [40] reported that subjects with generalized early onset periodontitis smoked more heavily (7.2 + 6.3 pack years) than those with localized forms of this disease (4.6 + 6.1 pack years). These findings suggest that not just the dosage but also the duration of the smoking habit is an important determinant of disease progression. Holm [41] investigated tooth loss in a Swedish population and identified young males who smoked more than 15 cigarettes per day to be at the most significant risk of tooth loss. The results of this study reported a relative risk of between 2.66 and 4.55 for light and heavy smokers respectively. A recent report from Calsina and colleagues [42] reported a 2.7 times greater probability to have established periodontitis in a study of Spanish adults over 20 years old. These investigators also observed a more significant effect in male patients and reported that the probability of having disease increased to 3.7 in those who had been smoking for 10 years or more. Hashim and colleagues [43] examined periodontal attachment loss in a cohort of 914 young adults and based on longitudinal smoking histories at ages 15, 18, 21 and 26 years determined that smokers had three times the likelihood to develop one or more sites with attachment loss of 4 mm or more. These investigators concluded that chronic exposure to smoking was a strong predictor of periodontal disease prevalence in young adults.

## Smoking and Alveolar Bone Loss

Arno and colleagues [44] previously reported a relationship between alveolar bone loss and tobacco consumption. The findings of Bergstrom and coworkers [14] when they investigated the relationship between cigarette smoking and bone loss in a group of dental hygienists were suggestive of an effect on alveolar bone that was independent of plaque levels. These investigators reported based on bitewing radiograph measurements that alveolar bone loss was more significant in smokers than non-smokers. They also reported that this relationship was age-related, suggesting that progression was more significant in younger smokers.

In a study population of referrals with aggressive periodontitis, Mullally and colleagues [31] demonstrated that the effects of smoking on alveolar bone loss are particularly evident in the maxillary arch. They observed periodontal destruction around canines and premolar teeth not classically part of the molar-incisor pattern as seen in localized juvenile periodontitis. In a study of generalised aggressive periodontitis Kamma and colleagues [34] also reported deeper periodontal pockets in the palatal aspect of maxillary teeth. The findings of these studies have been supported by clinical investigations that compared measurements of attachment loss in smokers and non-smokers [45]. This group also reported more significant periodontal destruction at the palatal aspect of maxillary teeth. The reports from other studies of older populations in which similar patterns of periodontitis have been described [46,47] and the high levels of tooth loss from the maxillary arch in smokers also support this view. The localised distribution of more severe periodontal destruction in this area lends weight to the suggestion that this might be the result of a local effect of cigarette smoke as the palatal tissues would be one of the first areas of contact during inhalation. The thermal effect of cigarette smoking on periodontal tissues has been studied but this investigation yielded inconclusive results [25]. Other studies have related smoking to reduced mineral content in bone [48] although any relationship between smoking, osteoporosis and periodontitis is not so clear.

The occurrence of furcation defects is generally uncommon even in young adults with severe periodontal destruction, however when they do occur the treatment is more complex and prognosis poorer. In a study of young adult regular attenders treated in general dental practices, Linden and Mullally [21], although reporting a low prevalence, noted that when furcation defects did occur these were exclusively affecting smokers. In a subsequent radiographic investigation of an older adult population of referrals to a specialist periodontal clinic it was reported that the prevalence of molar furcation defects among cigarette smokers was double that for a matched group of non-smokers [49].

## Smoking and Host Response

Nicotine metabolites can concentrate in the periodontium and their effects include the promotion of vasoconstriction, and the impairment of the functional activity of polymorphs and macrophages. The numbers of neutrophils in peripheral blood are also increased by tobacco use and their migration through capillary walls is impaired due also to paralysis of the cell membrane. Cigarette smoking has been demonstrated to activate the release of elastase, which has the capacity to cause tissue damage. The effect of this is particularly well displayed in lung diseases such as emphysema and in animal studies with elastase-deficient models, which do not develop emphysema when exposed to cigarette smoke. In addition there is an increased production of oxygen species, which can lower tissue levels of alpha-1 protease inhibitors so enabling elastase and other enzymes with potential for damaging tissue to remain unchecked at active sites [50,51]. The gingival crevicular fluid levels of functional elastase and that complexed with the inhibitor have been demonstrated to be lower in smokers than nonsmoking controls [52].

Much evidence on the effect of cigarette smoking on neutrophil activity suggests that these cells may accumulate at the site of inflamed periodontal tissues. As they may fail to migrate through the gingival crevice they can release their enzymes into the surrounding connective tissue therefore contributing directly to tissue destruction. Pauletto and colleagues [53] reported lower levels of both salivary elastase and neutrophils in the oral cavity in smokers with periodontitis. These researchers found that the number of neutrophils in nonsmoking periodontitis patients was twice that of the healthy group whereas the neutrophil count was only slightly elevated in the smoking periodontitis group. Their study demonstrated that oral elastase and neutrophil counts are lower in smokers compared with non-smokers with similar levels of periodontal disease. Their results also suggest that these values return to non-smoking levels after smoking cessation.

The role of the monocyte in the control of periodontal infection is to digest the antigen when the polymorph is unsuccessful. This results in the secretion of cytokines and release of chemotactic factors and those mediators, which can activate fibroblasts. Previous investigators have demonstrated an in vitro effect on monocyte secretion of bone resorbing inflammatory mediators by nicotine in response to lipopolysaccharide. Even moderate cigarette consumption can contribute to an increase in activated circulating monocytes and promote their adherence to endothelium. The role of cytokines has also been extensively examined in the pathogenesis of periodontitis. High levels of prostaglandin PGE2 have been associated with aggressive or early onset periodontitis and these elevated concentrations have been related to an increased responsiveness of circulating monocytes to bacterial challenge [54].

Mariggio and colleagues [55] reported that nicotine induced apoptotic modifications in Polymorphonuclear cells for up to 72 hours of culture time. These findings suggest nicotine exposure had a deleterious effect on the lifespan of neutrophils and modulation of their activities in respect to their crucial role in prevention of bacterial invasion of periodontal tissues. There appeared to be no effect on apoptosis of monocytes by nicotine exposure but these investigators did report that when stimulated by LPS monocytes exposed to nicotine had reduced IL-1_ release. This cytokine is proinflammatory and has been associated with osteoclast activity and alveolar bone resorption. Procoagulant activity expression is a function of monocytes related to limiting the spread of infections and its inhibition by nicotine is more evidence of the complexity of effect of tobacco on this part of the host defence mechanism.

In vitro studies have demonstrated that nicotine exposure can inhibit important antimicrobial activity such as production of superoxide anion and hydrogen peroxide [56]. Others have demonstrated depressed numbers of helper lymphocytes among cigarette smokers, which could effect both B cell function and antibody production [57-59].

## Smoking and Immunoglobulin G Production in Periodontal Disease

Immunoglobulin IgG2 is associated with reactions with bacterial toxins such as lipopolysaccharides. Infection with the periodontal pathogens *Actinobacillus actinomycetemcomitans *and *Porphyromonas gingivalis *have been associated with elevated serum IgG levels in early onset or aggressive periodontitis compared to periodontally healthy controls [60-62]. Lu and colleagues [63] reported higher serum IgG2 levels in subjects with localized early onset periodontitis compared to controls. It has been shown that tobacco smoking reduces serum Immunoglobulin G (IgG) levels. Previous studies demonstrated elevated serum levels of IgG2 in those with localized aggressive periodontitis compared with generalized aggressive periodontitis and healthy controls [30]. In these subjects it has also been shown that those who smoke are not clinically different from non-smokers with localized aggressive or juvenile periodontitis.

Quinn and colleagues [36] investigated the effect of smoking on Immunoglobulin G subclass in young adults with aggressive forms of periodontitis. Smoking was not found to depress serum levels of IgG2 in Black subjects except for those with generalized periodontal destruction. This group also had depressed serum IgG4 levels. On the other hand, young smokers with the generalized form of aggressive periodontitis have more extensive periodontal destruction than their non-smoking equivalents.

In a later study from the same group, [64] they examined the effects of smoking on serum IgG subclass concentrations in race-matched groups: Localized juvenile periodontitis, Generalized aggressive periodontitis, and age-matched periodontally healthy controls. These investigators used serum cotinine levels to validate smoking status, and radial immunodiffusion to determine serum IgG subclass concentrations. From their data they concluded that the effects of smoking were selective with respect to both IgG subclass and race. They found that smoking did not effect the concentration of IgG1 or IgG3 in Black or White subjects. It was however, associated with depressed serum IgG2 concentrations in both periodontally healthy White subjects and those with generalized aggressive periodontitis. Their data also supported a relationship between the periodontal diagnosis and IgG levels. This was evidenced by the reduction of IgG2 and IgG4 in Black smokers with generalized disease whereas the levels of IgG2 and IgG4 in Blacks with localized disease and healthy controls did not appear to be reduced by smoking. These effects were not seen in Black smokers with healthy periodontal tissues or those with localized disease. Recent work by Albandar and colleagues [65] has provided further evidence for a relationship between serum antibody concentrations and race. However, these researchers could not find any significant differences between serum concentrations of IgG subclasses in subjects with various levels of periodontal disease.

Tangada and colleagues [35] reported that smoking depressed anti-A. actinomycetemcomitans IgG2 in Black American subjects with generalised aggressive periodontitis but had little effect on those with localized forms of these conditions. They suggest this as an explanation for the observation that smoking affects the periodontal status of subjects with generalized but has a less significant affect on those with localized forms of aggressive periodontitis. These immunological findings are mirrored by clinical observations of more extensive periodontitis among smokers than encountered in non-smoking counterparts.

## Smoking and Cytokines in Periodontitis in Young Adults

Many studies provide conflicting data on the potential mode of action of smoking particularly in regard to the effects on inflammatory mediators. For instance Boström [66] found that TNF_ levels are elevated in the GCF of smokers while Heasman [67] found PGE2 values to be similar in smokers and nonsmokers. Shirodaria and colleagues [68] found that IL-1_ levels were significantly reduced in smokers. This reduction was consistent in heavy smokers in spite of their IL-1 A genotype. The results of this study supported the view that any action of smoking was unlikely to be associated with the elevation of IL-1_ levels. Interleukin, IL-1_ has been demonstrated to have a role in gingivitis and has also been shown to stimulate bone resorption and inhibition of bone formation [69]. Its role in the stimulation of prostaglandin production and inductive effect on collagenase and other proteases has been described previously [70]. High levels of IL-1_ also have been related to periodontal disease activity. Other researchers have demonstrated a relationship between genetic polymorphisms for IL-1_ and increased susceptibility to both adult and early onset periodontitis. [71,72]. Albandar and colleagues [65] could not demonstrate significant differences in IL-1_ levels in subjects with early onset periodontitis and healthy controls. They did however observe ethnic variability and reported a trend of lower serum concentrations of this mediator in Blacks than either Hispanics or Whites. However in this study they did not establish the smoking status of the individuals studied. It would appear therefore that variation exists among different racial groups and populations regarding the influence of interleukin genotype on the development of periodontitis.

Other researchers have shown differences in the humoral and cell mediated immune systems with effects on the cytokine and adhesion molecule networks attributable to cigarette smoking. [66,73,74]. The migration of leucocytes reacting to infection is dependent upon the interaction of adhesion molecules located on the surface of these cells with ligand pairs on the endothelium of blood vessels. The immunoglobulin ICAM-1 is expressed by endothelial cells and some leucocytes and is involved in both the processes of adhesion and extravasation. Smoking has been shown to cause an increase in ICAM-1 expression in endothelial cells in vitro. Rezevandi and colleagues [75] examined ICAM-1 and the receptor E-selectin in gingival biopsies from matched smokers and non-smokers. Their data supported the view that the upregulation role of gingival vascular endothelium in the expression of these adhesion molecules in the inflammatory response was retained in smokers. They demonstrated that the gingivae of smokers had significantly lower levels of blood vessels at sites of inflammation than non-smokers with periodontitis. These investigators speculated that this might be a factor in the reduction of gingival bleeding and emigration of leucocytes observed in smokers. They also reported lower percentages of vessels expressing ICAM-1 in smokers than non-smokers, which they related to a systemic effect of tobacco smoking.

## Smoking and Periodontal Infections

Few studies have conclusively demonstrated any relevant microbiological changes in the periodontal tissues attributable to smoking. Zambon and colleagues [37], using self-reported smoking data, investigated the relationship between periodontal pathogens and cigarette consumption in a group of 1,426 subjects in New York State. Using indirect immunofluoresence microscopy to detect known periodontal pathogens, they reported that smokers had an increased risk of 2.3 times that of former or non-smokers to harbor *Bacteroides forsythus*. The relative risk for *B forsythus *infection increased with increasing tobacco consumption and heavy smokers were at an increased risk compared with light smokers. They also reported an increased risk for smokers to have subgingival infection with *Porphyromonas gingivalis *although this was not found to be statistically significant. In this same study the investigators found smokers were 3 times more likely to harbor *A. actinomycetemcomitans*.

Haffajee and Socransky [76] investigated the relationship between cigarette smoking and subgingival microbiota using checkerboard DNA hybridization. Although in this study they specifically excluded those with aggressive forms of periodontitis, their findings are relevant. They concluded that the major difference between smokers and non-smokers was in the prevalence of species (i.e., periodontal pathogens colonized a larger proportion of sites) rather than counts or proportions. The increased colonization seen among smokers was particularly evident at the shallower pockets, i.e., those less than 4 mm. In addition they reported that a higher percentage of sites were colonized by *B. forsythus *and *P. nigrescens *in maxillary than mandibular sites. Haffajee and Socransky [76] suggest that the greater severity of periodontal destruction seen in smokers could be explained by the increased colonization by potential pathogens at more sites, which would in turn enhance the risk of further breakdown. Although unable to offer a precise explanation for this colonization pattern, these investigators suggest that a combination of local effects from cigarette smoking on the pathogens environment and a deleterious effect on the host response are important factors. Using cultivable flora techniques, Kamma and colleagues [34] investigated the effects of smoking in subjects with aggressive periodontitis. They reported that smokers harbored greater numbers of strict anaerobes, especially *Porphyromonas gingivalis, Bacteroides forsythus*, and *Eikenella corrodens *compared to non-smoking subjects.

Darby and colleagues [77] investigated the relationship between cigarette smoking and the prevalence of periodontal pathogens using polymerase chain reaction techniques. In this study, which included equal numbers of smoking and non-smoking subjects with generalised aggressive periodontitis, the investigators could find no significant differences in the occurrence of any of the pathogenic species which included *P. gingivalis, P. intermedia, B. forsythus*, *A. actinomycetemcomitans *and *T. denticola*.

## Smoking and Response to Periodontal Therapy

Preber and Bergstrom [46] reported that smokers did not respond as well as non-smokers to non-surgical therapy. Ah and colleagues [78] and Kaldahl and colleagues [79], who reported less probing depth reduction and attachment gain in smokers who had been treated by periodontal surgery, corroborated this finding that smokers were poor candidates for successful periodontal care. Kamma and Baehni [80] reported that smoking was found to have significant predictive value on future attachment loss in a five-year follow up of 25 young adults diagnosed with early-onset periodontitis who had been receiving regular periodontal maintenance care.

Boström and colleagues [66] also found less bone gain over a five-year study period. Mc Guire and Nunn [81] found double the risk of tooth loss in smokers undergoing maintenance periodontal care over a five-year period. Preber and colleagues [82] reported no difference with regard to elimination of microflora after non-surgical treatment. Several investigators reported that eradication of periodontal pathogens such as *A. actinomycetemcomitans *and *P. gingivalis *was more difficult after non-surgical periodontal treatment. Mooney and colleagues [83] investigated antibody titers to five periodontal pathogens in subjects with generalized aggressive periodontitis. His group found no statistically significant differences in untreated subjects but reported lower values for *Actinobacillus actinomycetemcomitans, Porphyromonas gingivalis *and *Treponema denticola *in periodontal patients receiving maintenance care who were smokers than non-smokers. These authors concluded that smoking might interrupt immune maturation subsequent to periodontal treatment.

Mosely and colleagues [84] demonstrated that cigarette smoking impaired re-vascularisation and Tipton and Dabbous [85] reported an inhibition of collagen and fibronectin production associated with smoking. Fibroblasts can bind and internalize nicotine resulting in high intracellular levels, which may interfere with normal cellular function [86]. James and colleagues [87] investigated the in vitro effect of nicotine on fibroblast activity. They found that it inhibited attachment and growth of periodontal ligament fibroblasts. The results of these studies all indicate that smoking has a deleterious effect on wound healing and may help to explain why smokers respond less favourably to periodontal therapy.

## Smoking Cessation in Young Adults

In a 10-year prospective study Bergstrom and colleagues [88] have demonstrated that periodontal stability can be achieved in former smokers whereas chronic smokers continued to suffer loss of alveolar bone height. This suggests that smoking cessation is beneficial to periodontal health although it should also be noted that the past effects of smoking such as periodontal attachment loss are irreversible. Authors of a recent epidemiological investigation from the United States reported a sharp decline in teenage smoking in 2002 following a peak of juvenile smoking observed in the mid 1990's [89]. This effect has been related to a number of apparently successful campaigns to counteract the perception of smoking among adolescents. Most adolescent smokers are addicted to nicotine and adolescent quitters suffer relapse rates, which are similar to adults. Prevention programs can reduce adolescent smoking and it is generally agreed that if kept tobacco free in youth most people will never start.

The dental profession is well placed to deliver smoking cessation advice to young adults given that while attendance at a doctor's surgery may be infrequent in this age group, regular dental visits are common. The incorporation of training in smoking cessation advice into the education of dentists and dental hygienists is now commonplace. However there still exists a significant need to address the problem of cigarette smoking in the overall management of young patients with aggressive forms of periodontal disease. The effects of smoking appear to be most significant among those with the generalized forms of aggressive periodontitis. The prevalence of cigarette smoking in this group has been reported to be as high as 70% [31]. These individuals should be a priority group for cessation counseling. Considering that most cigarette smokers start as teenagers it is not inconceivable that by their mid to late 20s that individuals may have experienced more than a decade of smoking exposure. As previously reported by Hashim and colleagues [43] smoking at this stage in life increases the likelihood for significant periodontal attachment loss, which in this study was clinically evident among a cohort of 26-year-olds.

In a review on health promotion and behavioral approaches in the prevention of periodontal diseases, Kallio [90] summarizes the chairside and societal options for the dental profession. The suggested chairside activities include a concise smoking history and offer of smoking cessation advice, consideration of the effects of family and peer group on the patient's behavior and the distribution of written information. A recent Australian study evaluated the patient's view of smoking cessation by dentists and reported that less than one third of smokers would be encouraged to quit if advised by their dentist [91]. However these investigators did report that 73% of their sample would expect their dentist to be interested in their smoking status and 64% expected to be advised about the effects of smoking on their oral health. Just 35% of this Australian sample had any recollection of their dentist giving them cessation advice. Rikard-Bell's research group and others have demonstrated a reluctance of dentists to become fully committed to smoking cessation counseling, although their results dispel any fears of patients becoming alienated by receiving such advice. They do point out, as do others, the suitability of dentists to provide these services on account of the frequency of dental visits. This study did not include any reference to periodontal disease and although 43% of the study population was under 40 years of age no detail is given on their periodontal status. One might speculate that smoking cessation advice in a periodontal setting would have more impact on the recipients on account of its relevance to the disease pathogenesis. The strategy of health promotion based on common risk factors as outlined by Kallio [90] may be of some benefit in expanding the relevance of smoking cessation to oral health and therefore increase the confidence of patients in the role of the dental team in counseling.

While there may be some reluctance on the part of the general dentists to become involved in the face-to-face activity of smoking cessation advice there is evidence that they have a role in interacting with other health agencies. The involvement of dental professionals in consort with other health care workers and administrators in the development of school policies on smoking bans has been advocated as an example of an effective community based interaction. One youth intervention study [92] reported 22% less lifetime cigarette consumption in the test group compared with the controls over a 15-year period.

## Conclusion

While the precise mechanisms whereby cigarette smoking can exert an effect on periodontal tissues are not completely understood it is clear that it is still the most significant preventable risk factor for periodontitis. Its effects are related to the duration and number of cigarettes consumed. The smoking status of the family members may also be relevant both in regard to behavioral influences and the potential consequence of passive smoking. This latter area is in need of further research. Besides having a range of systemic effects which can alter the host response cigarette smoking would also appear to have considerable local effects which may account for the premature establishment of the disease process in susceptible young adults.

Epidemiological studies have shown that aggressive or early-onset periodontitis might affect less than 1% of most developed populations but can be more prevalent in poorer nations, as Albandar and colleagues have reported from a study in Uganda [12]. As legislative conditions reduce the potential market for tobacco sales in the U.S. and Europe, tobacco companies appear to be targeting the less developed nations for their replacement markets. For these reasons one might expect that developing nations would experience an increase in the prevalence of severe periodontal conditions including aggressive periodontitis in line with the observations made previously by Hujoel [5].

While this review has primarily examined the effects attributed to nicotine and its metabolites it should be stated that other byproducts of cigarette smoke might also have an influence on the progression of periodontitis. It should also be noted that a significant genetic component has been identified in relation to aggressive periodontitis [13,68,72] and the combined interaction of cigarette smoking and various genetic polymorphisms might also contribute to disease status in young adults [81,93].

If we are to pursue our strategies of prevention, early detection of disease and prompt intervention then the dental profession should continue to target and educate our young patients about the effects of smoking on periodontal health. In this way dentistry will also be making a significant contribution to the general health and well being of our youth and future generations.

## Competing interests

The authors declare that they have no competing interests.
